# Motivation of university and non-university stakeholders to change medical education in Vietnam

**DOI:** 10.1186/1472-6920-9-49

**Published:** 2009-07-24

**Authors:** Luu Ngoc Hoat, Nguyen Lan Viet, GJ van der Wilt, J Broerse, EJ Ruitenberg, EP Wright

**Affiliations:** 1 Biostatistics and Medical Informatics Department, Faculty of Public Health, Hanoi Medical University, Dong Da, Hanoi, Vietnam; 2 Cardiovascular Department, Hanoi Medical University, Dong Da, Hanoi, Vietnam; 3 Faculty of Earth and Life Sciences, VU University Amsterdam, De Boelelaan 1085, 1081 HV Amsterdam, the Netherlands; 4 Science Communication Department, Faculty of Earth and Life Sciences, VU University Amsterdam, De Boelelaan 1085, 1081 HV Amsterdam, The Netherlands; 5 Athena Institute for Research on Communication and Innovation in Health and Life Sciences, Faculty of Earth and Life Sciences, VU University Amsterdam, De Boelelaan 1085, 1081 HV Amsterdam, The Netherlands; 6 Medical Committee Netherlands-Vietnam, Dong Da, Hanoi, Vietnam

## Abstract

**Background:**

Both university and non-university stakeholders should be involved in the process of curriculum development in medical schools, because all are concerned with the competencies of the graduates. That may be difficult unless appropriate strategies are used to motivate each stakeholder. From 1999 to 2006, eight medical schools in Vietnam worked together to change the curriculum and teaching for general medical students to make it more community oriented. This paper describes the factors that motivated the different stakeholders to participate in curriculum change and teaching in Vietnamese medical schools and the activities to address those factors and have sustainable contributions from all relevant stakeholders.

**Methods:**

Case study analysis of contributions to the change process, using reports, interviews, focus group discussions and surveys and based on Herzberg's Motivation Theory to analyze involvement of different stakeholders.

**Results:**

Different stakeholders were motivated by selected activities, such as providing opportunities for non-university stakeholders to share their opinions, organizing interactions among university stakeholders, stimulating both bottom-up and top-down inputs, focusing on learning from each other, and emphasizing self-motivation factors.

**Conclusion:**

The Herzberg Motivation theory helped to identify suitable approaches to ensure that teaching topics, materials and assessment methods more closely reflected the health care needs of the community. Other medical schools undertaking a reform process may learn from this experience.

## Background

Medical schools around the world have started to look outside their walls for evidence to support the changes needed to serve the public appropriately as patterns of health and disease change [1-3]. Communities also recognize that universities can contribute to community health [4]. Murray (1995) stated that medical education should reflect the school's responsibility to the community [5]. He found that 63% of 95 medical schools in North America tried to identify community needs and increase public involvement in their planning. The interaction between medical schools and various stakeholders may be even more important when health problems are increasingly complex in settings with limited resources and fragmented health systems as in Vietnam. Hays argued that stakeholders outside the university need to be involved in development of medical education, to build in accountability to the demands from society and to prepare graduates to work in communities [6].

The process by which a curriculum is designed and implemented can be executed in different ways. A traditional way is to have a vision developed by a small core team of professionals who translate that vision into the new curriculum and supervise the implementation by the training institutions [7,8]. Another approach is to set up a process of interactions among the different stakeholders and the medical schools for both the design and the implementation of the curriculum. The assumption in this approach is that such interactions are needed to produce a design that balances the training capacities of the schools and societal needs, and at the same time motivates the teachers to implement the curriculum [7,9]. The latter approach is especially relevant if health problems are becoming increasingly complex and changing rapidly. However, involving and motivating the different stakeholders is also costly and complicated in practice. Consequently, achievements of many initiatives have been limited [9]. It is important to identify the strategies and activities that will stimulate and encourage the involvement of a wide range of stakeholders to ensure that the final product, the curriculum, is appropriate to the needs of the society.

In this paper we analyse a process of change in Vietnam that has been quite successful in realizing a sustainable and profound change in medical education. We specifically look firstly at which stakeholders were involved in the process, where, when and how, and secondly, at the motivation of the different stakeholders resulting from the strategies applied by the project management. Motivation is analyzed and described according to Herzberg's Theory of Motivation, one of several theories that have been developed over the past decades to explain motivation for management; it is based on Maslow's Hierarchy of Needs [10].

In Vietnam, the political and socio-economic environment has changed considerably since 1986, when open-door policies and market mechanisms were introduced. At the same time, however, national policy has demanded more community-oriented doctors, thereby creating challenges for the traditional hospital-oriented curriculum. These changes have been strong push factors for the start of a change process in Vietnamese medical education since the early 90s. The process aimed at a comprehensive renovation of the medical curriculum, to make it more community-oriented. First school leaders and key teachers and later, all teachers and students along with a range of stakeholders from outside the university had to be motivated to join the efforts to review and revise the curriculum.

### Herzberg's Theory of motivation

**Figure 1:**
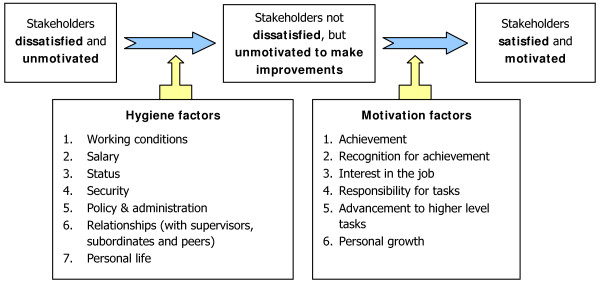
**Roles of hygiene and motivation factors according to Herzberg's Theory of Motivation**. * Source: Adapted from

Herzberg's Theory of Motivation was developed to describe the motivation of employees in an organization to deliver a higher performance [11]. According to Herzberg, what makes people work well can be distinguished as affecting either satisfaction or motivation. People who are not satisfied and not motivated will not work well, and people who are not dissatisfied but also not motivated will work well enough in the current situation but will not be active to change it. Herzberg separated the factors into two groups. One the one hand, he identified 'hygiene' factors, which determine the level of satisfaction (such as working conditions, salary, status, security, and relationships). These factors are largely "extrinsic", or external to the nature of the job itself. In contrast, the 'motivator' factors affect the person's willingness to improve performance (such as achievement, recognition, job interest, responsibility, advancement and personal growth). These factors are associated with job content and are "intrinsic", or found within each individual. The theory implies that if motivators are not present in a job, a person will not necessarily be dissatisfied, but will not be willing to participate in a process of change. Herzberg's theory is summarized in Figure 1.

This model seemed to be applicable in the Vietnamese context and helped us to analyse our case. In line with Herzberg's theory, we identified satisfiers and motivators that the project created to involve and motivate different stakeholders during the process of change.

## Methods

This study focused on the phase of the change process in which more stakeholders were involved and more change was planned; higher motivation levels were needed from a range of actors with different roles. It was a long process of change with the participation of thousands of teachers and students from eight medical schools in Vietnam and many other stakeholders outside the medical schools over a period of more than five years. It was, therefore, not practical to conduct a single survey using specific tools aimed at collecting data on the elements of Hertzberg's model. Using the case study evaluation approach, we could review, select and analyze the data from different studies along with reports and documents relevant to the model to answer the question of how the university and non-university stakeholders were involved and motivated to contribute to the success and sustainability of the curriculum change process.

The three main sources of the data reviewed were:

1. *Surveys*, using questionnaires, interviews and focus group discussions as listed in Table 1. These surveys were conducted to answer a range of research questions including those addressed in this paper; they have been published separately with a focus on their main topic (see Table 1) [12-17]. Details on the methods and results of each separate study can be found in the other publications. In this paper, we used only the data related to our research question.

**Table 1:** Sources of survey data for this study

1.	Study on the development of detailed learning objectives for six-year general medical students to produce the KAS book (a compendium of the learning objectives based on the knowledge, attitudes and skills graduates will need to address medical problems) with contribution of 913 teachers from eight schools and many other university and non university stakeholders [12]. A case study presentation method was used to evaluate the process and lessons learned from the use of a participatory approach to involve and motivate different stakeholders.
2.	Structured-questionnaire survey and 24 focus group discussions (FGD) among 797 recently graduated doctors practicing in the field to contribute feedback for KAS developed by teachers [13]. Among other issues they were asked about their learning during their medical studies.

3.	Survey among 1,136 sixth-year students from eight schools contributed feedback for KAS developed by teachers using a structured questionnaire [14]. Questions included topics of learning and providing feedback to teachers.

4.	Key informant interviews and FGD among 325 employers, local authorities, patients, relatives and recently graduated doctors during KAS survey (unpublished project report). The non-university stakeholders gave comments and suggestions to contribute to the improvement of training in medical schools.

5.	Interviews and FGD among 144 local health staff who acted as preceptors in field teaching (FT) for medical schools; interviews among 300 community members and 12 FGD with local authorities in two FT sites; questionnaire-based feedback survey among 240 students just returned from FT sites [15]. Questions and topics included the role of the health workers in teaching and medical education.

6.	Study on perceptions of 600 students in two medical schools on their learning environment after interventions of the project, using a standardized questionnaire, according to Roff [16,17], (Giang et al, in preparation)

2. *Project documents*, such as reports of meetings, workshops, inter-school assessments, project evaluation, and annual reports. These included reports on regular self-evaluation and supervision according to a standard checklist.

3. *Participatory observation *of the first author (who acted as project coordinator) and the last author (who acted as technical adviser), using direct observation, informal dialogues during meetings and workshops, and formal meetings with different stakeholders during supervision and assessment visits to the medical schools. Although participatory observation may lead to subjective and biased data, it has the advantage of providing highly rich data that would remain hidden to external researchers.

To improve the validity of the data, triangulation was applied among stakeholders and among data collection methods.

## Results

**Figure 2:**
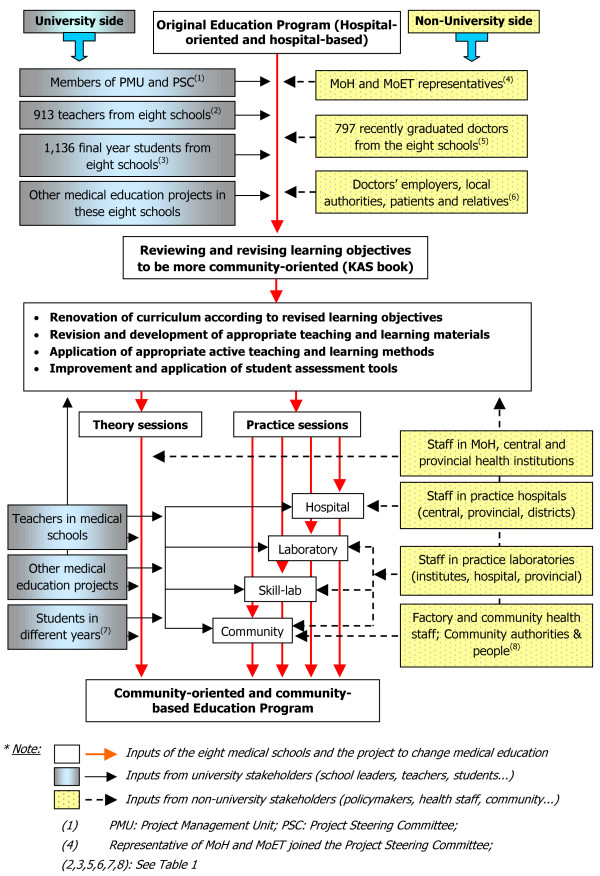
**Process of change and contributions from stakeholders**.

The medical schools wanted to change from a traditional hospital-oriented curriculum to more community-oriented education. That meant that it was necessary to involve stakeholders not only from within the universities, but also stakeholders outside the universities, to ensure that the changes brought medical education closer to the needs of society. Figure 2 shows which stakeholders were involved, and where and when they were involved.

There were two points at which the contributions of a wide range of stakeholders were especially important. The first was changing the learning objectives (formulated in a reference text called the Knowledge, Attitudes and Skills or KAS book) to ensure that they fit with the needs of relevant stakeholders, including the community. In this step, stakeholder involvement was not continuous; they were involved when the learning objectives needed to be updated. The following step was revising and implementing the curriculum based on learning objectives. In this step stakeholders needed to be involved more regularly and continuously, especially with respect to the daily teaching according to the curriculum. Therefore strategies to involve and motivate stakeholders depended not only on the kind of stakeholders, but also on where and when in the renovation cycle they were involved.

In the following sections, we describe the strategies that were applied to translate the various hygiene and motivation factors into practice, according to the types of stakeholders, either outside the university or within it.

### Non-University stakeholders

#### Ministry representatives

The Ministries of Health (MoH) and of Education and Training (MoET) are mandated to oversee curriculum development. MoET is responsible for promulgating the curriculum for all higher educational institutions, including medical education, while the content of the medical curriculum is the responsibility of and proposed by MoH. When we asked teachers or leaders of medical schools whether medical curriculum change would be feasible through the project, many considered it highly unlikely, as illustrated by the remark of a leader of a medical education project at a meeting on the scope of the project:

"The idea to change the curriculum of eight medical schools to community-oriented teaching is very necessary, but too ambitious; changing curriculum is only decided by MoH and MoET, not the university. We should focus on modest things like changing FT."

Also the International Senior Technical Advisor wrote in the report on the first international school visit:

"Many teachers and department leaders said that the project ideas are very appropriate and contribute to the needs of the schools; without the project, they still have to do that work. But many teachers also said it is difficult to do; the reason they often gave is that MoH and MoET will not allow it."

It was, thus, of critical importance to involve representatives of the MoH and MoET in the process, to use their authority to facilitate change. The project's management therefore invited the two Ministries to sit on the Project Steering Committee (PSC), which also included representatives of the power structure in the eight schools. The task of the PSC was to advise on the planning and implementation of the change process. The main reason for the representatives of MoH and MoET to accept the invitation to join the PSC was that they could see the project would provide support for work that they already wanted to do. In an inter-school workshop involving more than 200 heads of departments from the eight schools, the PSC member from MoH stated:

"Before this project, we had a committee with leaders of medical schools and a few senior teachers to propose the curriculum. Their draft was circulated among MOH departments for comment then sent to MoET for approval. Now, with support from the project, we are happy that the new curriculum will have inputs from other stakeholders."

These statements, made in the presence of key departmental leaders who had never had a chance to participate in curriculum development, created a ground-breaking moment that motivated participation in the interventions that followed. In addition, the project support provided opportunities for the Ministry to communicate more frequently, more widely and more deeply with the other stakeholders, especially those in the universities. Key motivators were job interest, achievement and responsibility, while important satisfiers were better health services with better trained doctors.

#### Health service providers

Before this project, health service providers were not involved in curriculum development although they could be asked to contribute to practical teaching. At the beginning of the project, health service managers were asked their ideas on the skills they required from new graduates joining their services. The Head of a District Health Centre stated:

"I recruit medical doctors graduating from different schools. I feel that training in medical schools has changed very slowly compared to changes in society and disease patterns. Many students have good knowledge and some high tech skills, but they lack common knowledge and skills that would help them deal with their daily duties. Most lack skills in planning, management and decision-making. When they come to work for us, we have to train them for six months before they work well."

These remarks illustrated the need for consultation with future employers. Later surveys provided similar critical views from other employers:

"Many young doctors assigned to district or preventive health centres don't focus on their work, mainly because they feel they lack knowledge and skills to work in those areas, and their contributions are less recognizable than in curative care."

(Head of district health centre)

*"Besides their many good qualities, we think that graduates need training to work more independently; they should understand more about community wisdom and involvement. They lack skills to explain to patients and families. They need more knowledge on planning and management, teamwork, and presenting their ideas to supervisors or local authorities. They lack report-writing and computer skills*.

(Vice-Director, provincial health bureau)

Their comments resulted in adjustments to the list of skills required of graduates that had been prepared by the teachers based on their own experience.

The health service leaders were highly motivated to contribute both because they needed better-trained doctors in order to deliver better care, and because the project consulted them, respecting their understanding and experience, and gave them more responsibility in sharing the design of the new curriculum.

#### Part-time teachers from hospitals and other institutions

The big hospitals, research institutes and MoH have many staff with a high level of training, with master and PhD degrees, who often have extensive practical experience, including management of health programs and facilities. Previous to the curriculum reform process, these experts were involved only in specific teaching sessions, but not in the rest of the work related to teaching. The project encouraged each department to invite staff from appropriate health institutions to join in learning to develop new teaching/learning materials and about new teaching methods. This involvement not only benefited the medical schools but also the part-time teachers, as is illustrated by the following quote from a part-time teacher in Internal Medicine:

"I've worked in this hospital for over 20 years and have been invited to teach medical students for eight years. Before, the department just assigned the topics and asked me to teach. Then, I thought that was enough, that invited teachers don't need to know everything going on in the school. Now they invited us to join other activities, such as development of KAS, curriculum and teaching/learning materials in very participatory ways; this created opportunities for us to learn, share and contribute to teaching in the school. That really motivated us to contribute to teaching. Besides that we also would like to gain enough teaching hours to be promoted as professors in the university."

These part-time teachers reported feeling proud to be invited to teach and to join other departmental activities, such as developing teaching materials. They appreciated the opportunities it gave them to learn about new developments, to share experience and to contribute to teaching future doctors. Their motivators were: recognition by students and teachers, new responsibilities, advancement and personal growth (especially towards professorship). They also received small financial incentives. Satisfiers included the increased job security when they were better trained and better relationships inside and outside their institutions.

#### Local Field Teaching preceptors

Community-based teaching is a very important aspect in community-oriented education and considerable attention was devoted to this part of the curriculum. Before the project, the role of health staff in field teaching (FT) had been limited, as illustrated by the remark of a doctor from a district hospital:

"Teachers from the university came to teach at our hospital. They did not involve us in the teaching, just moved their teaching to our hospital. There was no clear role for us."

To overcome the shortage of school teachers to joint FT and to make best use of the potential contributions of experienced local health staff, each school recruited local health staff to be trained as FT preceptors. Ranking of difficulties in FT identified two key issues: training of field preceptors and having them appointed to make teaching officially part of their work, not an additional burden.

"When you bring students, it is difficult for us to find time to teach them, it's not part of our official job, only unofficial extra work. If you can arrange for us to be invited as part-time teachers, we will be able to work more and better with the students."

(A health staff at district level)

The interventions included official invitations for local health staff to act as teachers during field periods, which improved the situation considerably. The preceptors were officially "recognized" and appreciated by teachers, students, their colleagues and communities.

The main motivating factors for field preceptors were the valued opportunities to share experience with students and teachers, and their pride in contributing to the training of future doctors (recognition and responsibility). They also upgraded their knowledge and skills when the university trained them, and increased their self-study to gain confidence to guide students (achievement and personal growth) as illustrated by quotes from two doctors at district hospitals:

"Before, we did not have much encouragement to continuously improve our knowledge and skills. But now that we are officially invited to teach the students, we are eager to learn more and improve our capacity so that we can share our experience with the students."

"Before if there were no patients for the students to see, I was not confident to discuss theory with them, but after training by university teachers, I can do that."

This also helped them in their routine work. Extra payment was less important than the other motivation factors. With respect to satisfiers, especially better working conditions, improved job security due to training and better relationships on the job and with schools were identified.

#### Community leaders and members

Given that community members can be considered the end users of medical education, obtaining insight into their health care needs is highly relevant in community-oriented education. Before this curriculum reform process started, they had little opportunity to contribute to medical education.

During a community consultation survey at the beginning of the project, community leaders, members and patients were asked to give comments about doctors. They were very motivated to do so; they felt proud, respected and appreciated and were happy to share their ideas:

"New doctors lack social knowledge. They are often not confident to negotiate with local authorities or to give health education to community people. They also find it difficult to work independently outside their hospital."

(Leader of a city People's Committee)

*"Doctors should be trained to work more independently at the district and commune level, where they often lack supervision. They should know more about laws and policies and they need to know more about management and health economics (how to allocate their limited budget). They should be trained more on communication skills, public speaking, and how to collect, summarize and analyze information"*.

(Leader of a district People's Committee)

"Young doctors have good medical knowledge, enthusiasm and provide good services. Patients find them easy to contact and ask questions. However, some of them could not understand well the situation and life of local people, especially in the countryside."

(Patient in district hospital)

People in two communities that hosted field teaching were asked their views and were positive about it. Most (80%) were willing to accept students in their homes during field periods; 30% had already done so. Community inhabitants and leaders recognized advantages in the students' visits – they could share life experience and health problems with them. The presence of students and teachers along with capacity strengthening for local health staff resulted in better health care. They did suggest that better coordination, especially better identification of the priority problems to be addressed, would help them more. The presence of teachers and students increased satisfaction with health care both for local health workers and community members, while involving them in planning FT and student assessment further increased motivation.

### University stakeholders

#### School leaders

The rectors, deans and deputies of the eight schools led and supported the process of change. These leaders were all motivated, especially in the early years, by the encouragement of the first Project Director, a famous surgeon and politician who provided a strong role model for the other school leaders to follow. The Deans of all eight schools signed a Memorandum of Understanding to confirm their commitment to participate in the change to more community-oriented teaching in medical schools:

*"Community-oriented teaching and application of active methods are needs and tasks of our medical schools and concerns of the ministries. The Dean Board of each school is responsible to mobilize resources to implement these tasks. The eight schools should form a network to help each other review and revise the curriculum to be community-oriented. This creates good opportunities to increase the relationships among the schools." *(The first article of the MoU)

During the final inter-school assessment visit to the eight schools, all leaders expressed their recognition that the project supported the work they had to do anyway, but also facilitated, expedited and improved it. The project increased their satisfaction, their performance improved and they achieved a better working environment, with more facilities and better teachers. Their interpersonal relationships increased and they were motivated by the recognition and appreciation they received when their schools improved.

"The way of working has changed. Before the Deans and Heads told staff how to do things. [The project] has made a breakthrough, a new way of doing things."

(Dean, medical school)

"We recognized the need to change but the project made it faster, provided resources. It changed teachers' attitudes to how students learn. We have gone from teacher-centred to student-centred. This is a major contribution."

(Deputy-dean, medical school)

This program provided a framework for new projects, which could contribute more effectively to the systematic improvement of teaching in the schools.

"Our project helps all eight medical schools to change the way of working and methods of teaching/learning. It did not support departments individually, but it created a framework for other projects, like a coat-rack for others to hang clothes in an orderly way."

(Former Dean, medical school)

Linking the project activities of the eight schools to the PSC with its ministry representatives meant that the products were accepted as official, as indicated by the first Director of the project:

"I introduced our KAS book at a medical education conference with MoH for leaders of all medical schools and international representatives. After my presentation, the Head of Science and Training Department said that MoH would consider the KAS book as a formal document presenting detailed learning objectives for general medical students."

#### Teachers

The teachers have traditionally been responsible for both developing and using teaching and learning materials (TLM) and student assessment tools. The project not only enabled them to perform this task better, but also provided them opportunities to work in new ways and to get inputs from more sources to provide a broader evidence base for their materials.

The eight medical schools are located in different regions; each school has its own catchment area, the provinces for which they are expected to provide trained medical doctors. Although they are all involved in training medical doctors, before the project, the department leaders and teachers of the schools seldom had opportunities to meet and share their concerns and experiences. Only the top leaders in the schools could meet regularly when invited by the ministries. During a number of workshops and exchange visits, the teachers could reach consensus on the revised learning objectives (KAS book) and a shared basic curriculum. That sharing was perceived by most as a very positive contribution to their own work; getting together motivated them to contribute more time and energy to improve medical education.

*"In the inter-school workshops, both younger and older teachers from both big and small schools could meet and have equal chances to contribute to the development of joint learning objectives and curriculum. They also had opportunities to meet and work with teachers from the same department in other schools to share their experiences and products. This sharing not only facilitated exchange of TLM and student assessment tools among schools (helping to solve shortages of TLM and tests in Vietnamese language especially in the smaller schools), but also motivated teaching staff to develop more and better materials and tests to exchange with other schools"*.

(Project Coordinator, during mid-term evaluation)

Within each department, the relationship among the teaching colleagues and their contributions to improving medical education also changed when they joined this process, as is illustrated by a quote from a lecturer of a Health Management Department during an inter-faculty visit:

"Before, only senior professors were considered suitable for writing textbooks. But now every teacher produced updated TLM for their own lessons. They can contribute to the department's textbooks. It is really a big change and it motivated us a lot. By having our names in the textbook we also gain points towards professorship."

The way of involving teachers in departmental work also motivated them to produce better quality teaching and TLM and gave more responsibility to the teachers:

"It's a good idea to assign each teacher to be responsible for teaching and developing materials for several lessons. They feel more responsible, they invest time and energy to update their knowledge to produce good materials. They have increased confidence to apply active teaching methods, which challenge especially young teachers because students ask more questions. Young teachers often have good foreign language and computer skills and can find updated information more easily than we can."

(Senior teacher, Paediatrics Department during inter-faculty visit)

To facilitate the development and approval of teaching and learning materials and assessment tests prepared by the teachers before they could be exchanged among the eight schools, the project provided detailed guidelines and checklists for every kind of TLM. The teachers could develop the materials then do a self-check before submitting the products to their department for review and approval. Each school also set up a Product Assessment Group that consisted of teachers with experience in both TLM development and the requirements for the new materials. Every product developed by teachers with project support was first reviewed by the leaders of their department to check the technical quality, and then reviewed by this Assessment Group. The teachers then received feedback and were asked to improve their products before they were accepted. This system stimulated and supported the teachers to develop good materials. The recognition by the department, the school and the other schools of their products was highly motivating for the teachers, as is illustrated by the remark of a young teacher (Department of Internal Medicine):

"With detailed guidelines and checklists, we found it easier to develop, check and improve TLM that meet with the school's new requirements. The checking by department leaders and then by the assessment group was not only to ensure good materials, but also to let teachers, especially the young ones, be more confident and enthusiastic to produce TLM. This process reduced considerably the number of inadequate materials, after the first round of checking. Therefore, it was really a good motivation for teachers to continue developing other TLM and to feel confident to do that."

The teachers' satisfaction increased (better policy and practice, better interpersonal relationships) while motivation factors (responsibility, recognition, advancement, job interest, personal growth and achievement) stimulated them to improve their performance.

#### Students

Students are, of course, the target group for medical education improvement. Therefore, the project took steps to get student feedback on the proposed and implemented changes during the project – students had never been involved in such a process before. Figure 2 shows that when the KAS book was developed, more than 1,100 final year students were consulted to check whether the leaning objectives were appropriate (see also Table 1). When the teachers began to use the new curriculum in the schools or in FT, using active methods, students in different years were consulted to get feedback on the new applications.

The motivation factors in this case were: teachers could get feedback and appreciation from students to continue to improve teaching/learning in schools, while students benefited from more appropriate learning objectives and curriculum; more active teachers using better methods, materials and assessment methods; and more objective student assessment.

The external evaluation at the end of the project found that students in all schools were very positive about active teaching methods and confirmed their use in most classes by that time.

"Why should we go to class when the teacher teaches us exactly what is written in the textbook? What we expect is to learn necessary and interesting things from the teacher's experience, and to ask questions we cannot answer ourselves. We highly appreciate teachers who have good active teaching methods and communication skills."

(3^rd ^year student)

Students were able to explain that active teaching is receiving background materials, being able to ask and answer questions in classes and clinics, and to participate in role plays or presentations. The survey of perceptions of students on their learning environment after interventions of the project (see Table 1) showed that the overall score (129/200) was high compared to some other countries, suggesting that the learning environment may be appropriate, and students seemed to be motivated to study (Giang, K.B., manuscript in preparation). Students recommended that more efficient planning of lessons would increase satisfaction and that more active teaching would increase student motivation. Active teaching and learning methods give students more responsibility for their learning, and more opportunities for personal growth and achievement. These were motivating factors for the students. Table 2 summarizes the results of our case study according to the two factor groups of Herzberg.

**Table 2:** Hygiene and motivation factors to involve different stakeholders in changing medical education in Vietnam

*Stakeholders*	*Hygiene factors*	*Motivation factors*
**1. Non-University stakeholders**		

1.1. MoH and MoET representatives	+ Better health services with better-trained doctors	+ Job interest and responsibility + Achievement and recognition

1.2. Leaders of health services	+ Better care provided with better doctors in future + Reduced extra work to train new doctors	+ Recognition and appreciation + Responsibility when they are involved in organizing and teaching

1.3. Part – time teachers from hospitals and other institutions	+ Job security when they were trained better + Better relationships inside and outside their institution	+ Recognition and responsibility + Advancement, personal growth, + Future professorship + Financial incentives (not important)

1.4. Local FT preceptors	+ Better working conditions + Job security with added training + Better relationships on job and with schools	+ Recognition and appreciation + Responsibility in FT + Achievement and personal growth + Financial incentives (not important)

1.5. Community leaders and members	+ Better and healthier living conditions/services + Better relationships with schools and students	+ Recognition and appreciation + Responsibility in FT + Personal growth (through health education and services)

**2. University stakeholders**		

2.1. School leaders	+ Better working conditions + Satisfy school policies + Better inter-personal relationships	+ Responsibility and job interest + Recognition and appreciation + Achievement and personal growth + More school facilities and income

2.2. School teachers	+ Better teaching conditions + Support for assigned tasks + Job security + Better relationships in department, school and external	+ Responsibility and job interest + Recognition and appreciation + Achievement and personal growth + Financial and professorship incentives

2.3. Students	+ Better learning conditions and environment + Better relationships in and outside school	+ Personal growth and achievement + Recognition and appreciation + Responsibilities and advancement + Investment in future work

## Discussion

A curriculum can be designed, developed and implemented using different approaches, according to the needs and circumstances in each setting. In Vietnam, when the curriculum needed to be changed, traditionally a small core team, consisting of medical school leaders, training department leaders and a few medical teachers with experience in curriculum development was established by the MoH to participate in revising the old curriculum [8]. There were many limitations to this approach. Firstly, few of the teachers who implement the curriculum were involved and teaching methods were not considered. However, as pointed out by French [7], the classroom teacher is the crucial ingredient for the success of any educational innovation. Secondly, non-university stakeholders such as local authorities and lay community members are not medical experts but are the consumers of health care services and have vested interests in ensuring optimal health care for themselves, their families and their communities [9]. They can, therefore, make meaningful contributions to facilitate choices in medical education. With the support of a project, eight Vietnamese medical schools used a participatory approach to work with a wide range of stakeholders to change their curriculum and consequently the teaching and learning methods and materials.

It was complicated, costly, time consuming and can be tricky to motivate all of the stakeholders involved in the process, but it was quite successful with the application of a participatory approach. Although Herzberg's theory has made a wide impact on the field of job attitudes and motivation, it still has significant number of critics [18-20]. Among those critical of the use of this theory, Whitsett and Winslow suggested that many investigations were characterized by methodological flaws, misrepresentation of results and gross misunderstanding of the theory [21]. Herzberg's theory is widely cited in the business literature, but less often in education, especially for teachers [22]. When Bellott and Tutor conducted a study among elementary and secondary school teachers, they reported that salary was an important factor to motivate teachers, while according to Herzberg, money would be a hygiene factor, not a motivation one [23]. The motivation of medical teachers and of clinicians was compared by Schormair [24]. Among ten statements on the motivation of medical teachers to teach, financial incentive played a small role in comparison with other incentives, such as teacher of the year award or student rating. Motivation for practising clinicians to teach medical students was due to intrinsic issues such as altruism, intellectual satisfaction, personal skills and truth seeking; reasons for clinicians not to get involved in teaching included lack of involvement in course design, or a heavy clinical workload [25]. In Vietnam, the situation may be similar in some ways to other countries, but local regulations and culture may also influence the motivation. For example, medical schools can accept health professionals outside the university as teachers if they contribute enough to teaching students in their working location. The status conferred on the professionals by their association with the university can motivate non-university stakeholders to teach. A Vietnamese saying, "Salutation is more appreciated than a party." reflects the importance of respect and appreciation compared to material benefits. Indeed, community-level health staff in Vietnam were found to be motivated mainly by appreciation of employers, colleagues and the community, as well as job stability and training opportunities; financial issues were less important [26].

Looking at the activities that were applied to involve and motivate the different stakeholders, the following strategies appear to have been crucial.

Firstly, it was observed that *involving stakeholders outside the university*, such as health service staff, part-time teachers, FT preceptors and communities, actively in the process of curriculum development and implementation in itself contributed to their motivation. Many of them were pleased to contribute to forming future doctors. They felt flattered and proud that their expertise and opinions were appreciated by the university.

Secondly, there was a strong focus on *interaction between the different stakeholders*, e.g. between teachers and school leaders, part-time lecturers and FT preceptors, and between school leaders and the ministries. Opportunities for different stakeholders to meet, to discuss and to share medical education experiences and health care needs motivated all of them to work towards a better medical curriculum to provide good doctors. It was proposed by Schormair that reforms in medical education must consider the interaction between medical teachers and their students [24], but this project went beyond that interaction to promote a broader process of exchange.

Thirdly, the process combined *bottom-up and top-down *approaches. Government policy and the authority of the two Ministries and the school leaders were combined with contributions from many stakeholders who were not usually given a chance to express their views on the subject. Both fulltime and part-time teachers were motivated by respect for their inputs. Not involving them in curriculum development could result in poor implementation due to lack of motivation on the part of teachers [25], while excluding authorities would weaken the project with respect to realizing an impact on curriculum change, which would have discouraged school leaders and teachers.

*Learning *is motivating in itself, and that strategy was highly motivating especially for the part-time teachers, like clinicians, researchers, laboratory technicians and field preceptors. They received training in technical topics from the university teachers, but were also trained in teaching and learning methods. When the university teachers joined the students in the field or practice sites, the part-time teachers could also learn from participating in teaching for students, thereby strengthening their own capacity and providing additional motivation.

Last but not least, it is important to note that the project put a strong *emphasis on self-motivation factors *for stakeholders, like learning-by-doing or working for recognition and appreciation, while minimizing motivation by monetary incentives. The main factors influencing motivation for clinical teachers have been reported to be intrinsic issues, such as altruism, intellectual satisfaction and personal skill development [25]; the same appeared to apply here. According to Coleman, two components underlie this kind of motivation: altruism and personal gain. Altruism meant providing service to the community and repaying the system, while personal gains included improved knowledge, improved self-esteem and relationships [27].

Not every stakeholder was equally motivated; there were part-time teachers who could or would not find time to improve their capacity to teach the students and there were teachers who did not want to accept feedback from the students. A detailed discussion of their lack of response to the factors discussed here is beyond the scope of this paper but will be necessary to complete the picture.

One limitation of Herzberg's Theory of Motivation is that it was originally developed to manage staff motivation inside an organization [11], rather than motivation of a range of stakeholders from different organizations. That meant that several of the factors found in Herzberg's theory, especially hygiene factors like salary, job security, company policy and administration, would be less relevant for some of the stakeholders described here, especially those from outside the university. Therefore these factors were not included in Table 2. In this particular situation, a factor like 'relationships with colleagues' acted not only as a hygiene factor, as Herzberg found, but also as a motivator. The repeated mention of the opportunities to meet and exchange experience with colleagues from other medical schools during this participatory process showed the importance the teachers gave to such exchanges. Because Herzberg focused on the employees within an organization, the theory also may not have considered the factors that may motivate those who are outside the university to become involved in the cycle of change within the university.

An interesting question is whether this attention to motivation of various stakeholders contributed to sustainability of the results of the change process. There are indications that this is indeed the case. Many interventions that started with the support of this project can be expected to last, especially because many of the factors that motivated the stakeholders still remain when the project was completed, such as responsibility, recognition, advancement, job interest, personal growth and achievement. Although the financial incentives during the project period could play a role in motivating the stakeholders, such incentives would no longer be available when the project finished. Therefore, payment was not given for just participating in project activities; financial rewards focused on products and skills that could be used for a long time, such as the KAS book, joint curriculum, teaching/learning materials, student assessment tests and active teaching methods. The part-time teachers working outside the universities were trained and as described above, were motivated to continue to learn once they had become involved.

"You ask about sustainability of activities that this project supported for our university? I don't think there is any problem. Development of teaching, learning and assessment materials are activities we have to do anyway, so teachers have to update materials to teach and produce student assessment tools. Now the project already supported them to develop, why not use them?"

(Teacher, Internal Medicine department)

Collaboration among the different stakeholders was based on a win-win approach. For example, once teachers benefited from exchanging materials among identical departments in different schools and communicating with their colleagues about their teaching experience, they were motivated to continue producing and exchanging TLM in that network. Some of the project-funded opportunities, such as big inter-school workshops and regional study visits or conference attendance might be difficult to sustain giving the limited budgets of the MoH and the medical schools. One solution was to create a new project that will use advances in information technology to establish online forums, teleconferencing and distance learning to support continued networking activities among the various stakeholders.

## Conclusion

When stakeholders from outside as well as groups inside the university are involved in a process of curriculum change, the product may be more likely to be appropriate to the needs of the both students and society. Not all stakeholders, however, find it convenient to become involved. Table 2 summarizes the satisfaction and motivation factors that applied to different stakeholders involved in a process of change in eight medical schools in Vietnam. Important strategies to enhance satisfiers and motivators were: active involvement of stakeholders outside the university, both bottom-up and top-down inputs, a focus on learning, and an emphasis on self-motivation factors. Regular face-to-face interactions among stakeholders were also very motivating.

Other universities in Vietnam and elsewhere could use the lessons learned from this program and process to make greater efforts to establish a system that offers not only opportunities, but also advantages to stakeholders both inside and outside of the schools, to ensure that they can all contribute their ideas.

## Competing interests

The authors declare that they have no competing interests.

## Authors' contributions

LNH was the project coordinator; he guided and participated in design, implementation and analysis of the surveys that contributed to this paper. He also collected data during the inter-school supervisions and carried out statistical tests for surveys and researches and drafted the manuscript. NLV was the project director, who directed the research, contributed ideas during analysis and writing, and reviewed the draft versions of the paper. GJW, JB and EJR were involved in the study planning, interpretation of the data and drafting of the manuscript; EPW joined LNH in the design and data collection during collaboration with the several universities in the project, and contributed to the data analysis as well as the drafting and editing of the manuscript. All of the authors have read and approved the final manuscript.

## Pre-publication history

The pre-publication history for this paper can be accessed here:


